# “Many miles to go …”: a systematic review of the implementation of patient decision support interventions into routine clinical practice

**DOI:** 10.1186/1472-6947-13-S2-S14

**Published:** 2013-11-29

**Authors:** Glyn Elwyn, Isabelle Scholl, Caroline Tietbohl, Mala Mann, Adrian GK Edwards, Catharine Clay, France Légaré, Trudy van der Weijden, Carmen L Lewis, Richard M Wexler, Dominick L Frosch

**Affiliations:** 1 Cochrane Institute of Primary Care and Public Health, Cardiff University School of Medicine, Heath Park, CF14 4YS, UK; 2 Department of Medical Psychology, University Medical Center Hamburg-Eppendorf, Martinistr. 52, D - 20246 Hamburg, Germany; 3 Department of Health Services Research, Palo Alto Medical Foundation Research Institute, 795 El Camino Real, Palo Alto, California, 94301, USA; 4 Office of Professional Education and Outreach, The Dartmouth Institute of Health Policy and Clinical Practice, 46 Centerra Parkway, Suite 203, Lebanon, New Hampshire, 03766, USA; 5 Knowledge Transfer and Health Technology Assessment Research Group, Research Centre of Centre Hospitalier Universitaire de Québec, Hôpital Saint-François D'Assise, 10, rue de l’Espinay, Québec, QC, G1L 3L5, Canada; 6 Department of General Practice, School CAPHRI, Peter Debyeplein 1, 6229 HA, Maastricht, The Netherlands; 7 University of North Carolina, Campus Box 7110, Chapel Hill, North Carolina, 27599, USA; 8 Informed Medical Decisions Foundation, 40 Court Street, Suite 300, Boston, Massachusetts, 02108, USA

## Abstract

**Background:**

Two decades of research has established the positive effect of using patient-targeted decision support interventions: patients gain knowledge, greater understanding of probabilities and increased confidence in decisions. Yet, despite their efficacy, the effectiveness of these decision support interventions in routine practice has yet to be established; widespread adoption has not occurred. The aim of this review was to search for and analyze the findings of published peer-reviewed studies that investigated the success levels of strategies or methods where attempts were made to *implement* patient-targeted decision support interventions into routine clinical settings.

**Methods:**

An electronic search strategy was devised and adapted for the following databases: ASSIA, CINAHL, Embase, HMIC, Medline, Medline-in-process, OpenSIGLE, PsycINFO, Scopus, Social Services Abstracts, and the Web of Science. In addition, we used snowballing techniques. Studies were included after dual independent assessment.

**Results:**

After assessment, 5322 abstracts yielded 51 articles for consideration. After examining full-texts, 17 studies were included and subjected to data extraction. The approach used in all studies was one where clinicians and their staff used a referral model, asking eligible patients to use decision support. The results point to significant challenges to the implementation of patient decision support using this model, including indifference on the part of health care professionals. This indifference stemmed from a reported lack of confidence in the content of decision support interventions and concern about disruption to established workflows, ultimately contributing to organizational inertia regarding their adoption.

**Conclusions:**

It seems too early to make firm recommendations about how best to implement patient decision support into routine practice because approaches that use a ‘referral model’ consistently report difficulties. We sense that the underlying issues that militate against the use of patient decision support and, more generally, limit the adoption of shared decision making, are under-investigated and under-specified. Future reports from implementation studies could be improved by following guidelines, for example the SQUIRE proposals, and by adopting methods that would be able to go beyond the ‘barriers’ and ‘facilitators’ approach to understand more about the nature of professional and organizational resistance to these tools. The lack of incentives that reward the use of these interventions needs to be considered as a significant impediment.

## Background

The difficulty of translating knowledge into practice is well established and is a familiar phenomenon to researchers who promote the adoption of patient decision support interventions (DESIs) [1,2]. Two decades of research has established the positive effect of using these interventions; patients gain knowledge, better understanding of probabilities and increased confidence in decisions [3].

The policy context has gradually become much more supportive in recent years. In the US, the 2010 Affordable Care Act (US) [4] was explicit about the promotion of shared decision making (SDM) and the use of DESIs. Some states have passed legislation supporting their use [5]. Similarly in the UK, SDM has been at the center of policy developments [6] and investments have been made in the development of online DESIs [7]. Canada is supporting province-wide work in the use of DESIs in Saskatchewan [8]. Many other countries are alert to the benefits and are considering policy developments in this area [9].

Yet, despite these policy developments and the existence of over 80 randomized controlled trials (RCTs) that have demonstrated the efficacy of these interventions [3], their adoption into mainstream clinical practice has yet to be established, and their impact when used in routine workflows requires evaluation. There are reports of early implementation efforts in the field but many are not yet published in the peer-reviewed literature [10]. For over a decade, the Dartmouth-Hitchcock Medical Center in New Hampshire, has routinely provided many patients with DESIs (DVDs and booklets) through their Center for Shared Decision Making [11], and Group Health in Seattle has reported organization-wide adoption of DESIs for selected conditions [12,13]. However, these well-known settings remain isolated examples of adoption. Though there are many who develop and evaluate these tools in academic settings, no studies of sustained wide-scale adoption have been reported.

In 2006, Gravel described clinicians’ reluctance to use patient DESIs because they did not believe that they were applicable to their patients and clinical situations [1]. Légaré examined 6764 titles and abstracts and analyzed five RCTs [2], concluding that the promotion of SDM may depend on training health care professionals and the adoption of patient targeted DESIs [2]. A conceptual analysis using the normalization process model highlighted some of the intra-organizational issues that might underlie the difficulties that are being experienced [14].

The stimulus for this review arose from work being undertaken by the International Patient Decision Aid Standards (IPDAS) Collaboration, which has produced a checklist [15] and an instrument to assess the quality of these interventions [16]. The IPDAS Collaboration initiated a review of its quality dimensions in 2010. As part of this work, we wanted to know whether we could identify evidence to inform recommendations about how best to implement patient DESIs into practice. We wanted to reflect the increasing emphasis being given to delivery research encompassing implementation or improvement science [17]. Pronovost highlights an issue that is becoming of central importance for policy makers – to examine why interventions that have positive effects for patients under controlled conditions do not become established in routine settings [17]. To address this gap in knowledge, the aim of this review was to search for and analyze studies that investigated the success levels of strategies or methods where attempts were made to *implement* patient-targeted DESIs into routine clinical settings.

## Methods

We undertook a systematic review using the following definitions and approach to search, selection, and data processing.

### Definition of implementation

This review is focused on work designed *to implement* patient DESIs into routine clinical settings. We adopted the following definitions: “… implementation is the constellation of processes intended to get an intervention into use within an organization” [18] and “… implementation is the critical gateway between an organisational decision to adopt an intervention and the routine use of that intervention, i.e., the transition period in which targeted stakeholders become increasingly skillful, consistent and committed in their use of an intervention” [19]. We are aware that the nature of patient DESIs can vary [20]. We focus on the following types of DESIs in this review: 1) brief tools designed for use in synchronous encounters (face-to-face or mediated by other means) and 2) more extensive tools (booklet, video, DVD, or websites) that clinicians recommend patients to use, either before or after clinical encounters.

### Search strategy

An electronic strategy was devised in collaboration with an information scientist (MM) and adapted for the following databases (1947- 24 January 2012): ASSIA, CINAHL, Embase, HMIC, Medline, Medline-in-process, OpenSIGLE, PsycINFO, Scopus, Social Services Abstracts, and the ISI Web of Science (Science Citation Index Expanded, Social Science Citation Index, and ISI Proceedings); see the Additional file 1, Appendix 1. Specific author searches were performed on the following researchers: M. Holmes-Rovner, K. R. Sepucha, J. Belkora, D. Frosch and D. Stacey. In addition, we used a range of “snowballing” techniques to increase the sensitivity of the search, including reference list follow up, contact with subject experts, and searching the content tables of relevant journals. We used Google Scholar and also searched the King’s Fund website. Articles included in a review of strategies to implement SDM were also considered [2]. Research colleagues were alerted to the review using two electronic networks, e.g., the SDM listserve (n = 470), and the SDM Facebook group members (n = 346).

### Inclusion and exclusion criteria

Studies published in peer-reviewed journals were considered if they reported on the use of methods to promote the use of patient DESIs in routine practice. Studies were included if they assessed barriers to implementation and/or investigated the process of introducing organizations to the potential use of these interventions. RCTs that studied *implementation* strategies were included, provided their outcome measurements included assessments of whether these interventions became integrated into routines, at the system level or equivalent. All health care settings and all patient groups were considered, including systems where patients were directed to access DESIs by contacting a telephone call-center or using the web. No date or language restrictions were used. Studies were excluded if they did not attempt to implement DESIs in routine practice, if their sole aim was to measure the effect of DESIs at the patient level, or if they evaluated more general interventions to “activate” patients.

### Study identification, data extraction, and analysis

Search outputs were merged and duplicates were removed. Titles and abstracts were assessed independently by two reviewers and disagreement was resolved by discussion. Data extraction forms were piloted and adapted. Data from each publication were extracted, even if articles reported the same study. The following fields were used: study identifiers, study type (RCT, quasi-experimental, observational, quality improvement report, case study report, other), intervention or implementation strategy, research method, country and study setting, underpinning implementation conceptual framework, health care delivery funding model (general taxation, voluntary or private insurance, other), groups described (implementation group, professional group, patient group), study purpose, duration, funder, incentives for patients or professionals, organizational level (microsystem or team, department, institution), DESI type, point at which the DESI is introduced to the patient (e.g., before, during, or after the clinical encounter), method of distribution. Data were also extracted on implementation outcomes, including the number of patients who were eligible, referred to or provided with DESIs, used DESIs and were seen by a health professional after using DESIs. Finally, data about outcomes related to professionals and systems, e.g., views, barriers, and facilitators were extracted. Independent data extractions, completed by IS and CT, were compared and discussed. Disagreements resolved by discussion with GE. Each study was summarized, and a descriptive synthesis of the results was produced. Our stated goal was to provide a narrative review and we therefore did not set out to formally assess study quality.

### Assessment of implementation level

Each study was assessed independently by GE and IS and categorized according to the intervention described and the level of implementation achieved, using an adapted model of implementation; see Table 1[21,22]. Disagreements were resolved by discussion.

**Table 1:** Five stages of achieved implementation (adapted from Grol et al) [21,22]

Stage	Description	Criteria for assessment
1. Orientation	Awareness and interest in innovation.	Distribution of messages, key figures, and networks approached and informed.
2. Insight	Understanding and insight into implications for routines.	Provision of instruction materials using audit methods and performance feedback.
3. Acceptance	Positive attitude to change, positive intentions/decision to change.	Adaptation of innovation by target group, identification of resistance to change, involvement of key individuals, pilots and demonstration of feasibility, detection of barriers, and search for solutions.
4. Change	Actual adoption, try out change in practice, exploratory use, confirmation of value of change.	Provision of resources, support for skills training, redevelopment of processes, temporary resource support, inventory of barriers, and solution attempts.
5. Maintenance	New practice integrated into routines/routine use, new practice embedded in organization, sustainability over time.	Long-term monitoring, feedback and reminder systems, integration into routine pathways, provision of researches, and support from management.

## Results

### Studies included

**Figure 1:**
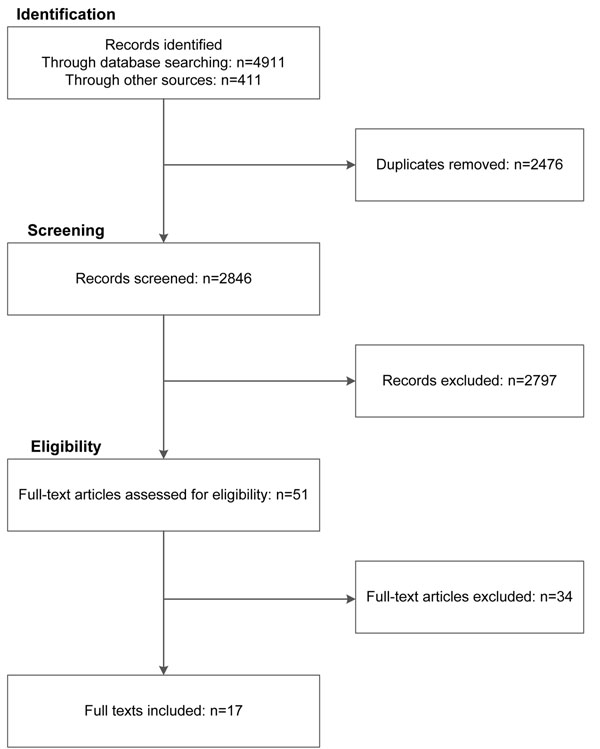
Flow diagram: Search outputs, study identification, and inclusion

Databases searches in July 2011 and January 2012 generated 4911 abstracts. Four hundred and eleven (411) additional abstracts were identified using other sources. After removing duplicates, 2848 abstracts remained. Examining Légaré’s review of interventions to implement SDM [2] did not lead to further study inclusion. After independent review by IS, CT, and GE, 51 studies were retained for further discussion by two raters (IS and GE). After examining full-text articles, 17 were retained for data extraction; see the flow diagram in Figure 1. A total of 34 studies were excluded at this stage. Further details of the studies excluded are provided in Table 2.

**Table 2:** Studies excluded after review of full-text articles

Reasons for exclusion (assessment of 51 full text articles)	Author, year, and study citation	Number of studies excluded
Intervention was not a DESI.	Belkora, 2005 [59]; Kotecha, 2009 [60].	2
Not an implementation study (i.e., primary aim was efficacy or other).	Bhavnani 2010 [61], Charles 2004 [62], Doran 2009 [63], Frosch 2008 [64], Graham 2007 [65], Hamann 2007 [66], Lewis 2008 [67], Ossebaard 2009 [68], Stacey 2009 [69], Stacey D, O'Connor 2003 [70], Thistlethwaite 2007 [71], Towle 2006 [72], Watson 2008 [73], Hirsch 2011 [74].	14
Article was an editorial, a model, a review, or had not been subjected to peer review.	Billings 2004 [75], Demilew 2004 [76], Holmes-Rovner 2007 [77], Lenert 2010 [78], Lewis 2009 [79], Légaré 2008 [80], Légaré 2010 [2], McCaffery 2007 [81], O'Connor 2005 [82], Pignone 2009 [83], Scott 1998 [84], Sepucha 2009 [85], Sepucha 2003 [86], Simmons 2010 [87], Wen 2010 [88], Wirrmann 2006 [89], Vandemheen 2011 [90].	17

### Overview of studies

The studies are summarized in Additional files 2 and 3, Tables S1 and S2. The majority of studies included used mixed methods (n = 11). Three studies used qualitative methods [23-25], and three relied on quantitative methods [26-28]. Eleven studies were based in the US and two in the UK [23,29]. Four studies were based in call-centers (three in Canada [27,30,31] and one in Australia [32]). Implementation was studied in both primary and secondary care settings, often involving multiple professions. Decision support for “screening” tests was mostly based in primary care or in internal medicine organizations. Clinical topics were varied, with several studies on breast and prostate cancer. Notwithstanding their common focus, there was significant diversity in both approach and evaluation. Eight of the 17 studies had been supported by the Informed Medical Decisions Foundation (IMDF).

### Conceptual frameworks

Of the 17 studies, few describe an explicit implementation framework as the basis of their evaluations. Stacey cites the Ottawa model of research use in four similar studies [27,30-32], a model based on knowledge translation [33]. Roger’s “theory of innovation diffusion” is cited by Feibelman [34]. Holmes-Rovner [35] and Belkora [36] use a logic model as the basis for their evaluations.

### Implementation strategies

Of the 17 studies, six were based on recruiting organizations at an institutional level and eight at a team or department level. Evaluation was often based on counts of the number of DESIs given to patients and counts of patient use based on follow-up surveys. Studies in nurse-led call-centers used training events and simulated patient callers to assess professional willingness to use patient DESIs.

Almost all of the studies used a “referral” model of DESI implementation, where patients were either sent the DESI by mail and asked to view it, or were directed to use the DESI (at home or in clinic) by either the clinician or another member of the clinical team. Most studies reported difficulties in operationalizing the referral model. One study compared different methods of delivering DESIs to patients eligible for a preventive-type decision (e.g., colorectal cancer screening) [26]. The authors found that systematic automated delivery was most efficient in reaching the greatest number of eligible patients, although it led to 20% of patients being inappropriately offered the intervention [26]. Irrespective of delivery mode, the patient viewing rate was estimated to be 25% of those sent out [26]. Belkora’s approach of getting pre-medical students to coach patients to list questions and use DESIs ahead of clinical encounters is a variant of the referral method, and relies on the identification of eligible patients ahead of encounters with clinicians [36,37]. All methods required organizational commitment. Only one study reported implementation costs, using estimates of the staff time used to identify patients [28].

### The existence of barriers

The dominant theme in a majority of the studies was the existence of barriers to efficient delivery and, therefore, implementation. Stacey [32], Feibelman [34], and Frosch [24] reported professionals’ attitudes and their call for more training in how to use decision support and undertake SDM [27,30-32]. There are also reports that clinicians may not trust or agree with the *content* of DESIs [23,34,38]. Some professionals were reported to hold the view that patients did not want decisional responsibility when facing difficult diagnoses [39] and that DESIs were in “competition” with other information designed for patients, suggesting that the intended aim of the DESIs, (i.e., to support patients in engaging in decisions), was not always understood [23,31,34].

Studies also reported that clinicians did not view the task of referring patients to use DESIs as part of their role, often citing competing demands and time pressure as the main reason why they could not incorporate this task into their usual practice [23,24,26,31,34,36-40]. As Bracket reports, when clinicians were responsible for identifying patients, distribution of DESIs failed because they were “distracted by other duties” [26]. Frosch [24] and Uy [25] describe two such studies, characterized essentially by implementation failure, particularly in organizations where team work was poor. One study illustrated this disinterest by using a modest financial incentive to encourage DESI distribution to patients; although effective while in operation, this strategy had no lasting impact as distribution ceased completely once the incentive ceiling had been met [25].

The studies demonstrate significant gaps between those patients who were deemed eligible, those who are successfully provided with the tools, and those who made use of them [26,28,34,38-40]. Patient-level measures were, for the most part, not reported and were not the focus of this review.

To overcome the problem of competing demands and low prioritization, system-based approaches were tested and found to be more successful [26,28,36,37]. However, system-based approaches rely on clinical problems where patients can be identified ahead of visits to the clinic. In situations where patients can potentially be identified ahead of clinic visits, logistical problems were reported. Mailing DESIs to patients will only be effective if patients use them. Brackett reported viewing rates of 25% [26], and, in a referral model, Uy reports 37% [25]. Inviting patients to view the DESIs in-clinic prior to a visit requires space, equipment, and a well-organized scheduling system. All require organizational commitment. Call-center settings also report organizational tension, notably a concern that call-handling efficiency might be disrupted by the adoption of decision support protocols [32].

### Facilitators

Some studies report factors that facilitated the use of DESIs. The provision of training and skills development for providers [30,31,35], and the identification of a clinical champion, especially in a leadership position, were important positive factors [25,40]. However, the most often cited predictor of success was the introduction of a system where eligible patients were systematically identified [26,40], or supported to use DESIs ahead of relevant clinical consultations [36,37]. In other words, methods of distribution that did not to rely on clinicians to initiate access to these tools proved to be the most effective by far.

### Levels of implementation achieved

The levels of DESI adoption achieved were generally framed by the studies as being “less than expected”. However, the studies did not explicitly report whether or not sustained use of DESIs had been achieved, although testing “feasibility” was often the primary aim and reported early stages in learning about the potential use of these tools. Nevertheless, the implicit goal in most studies was to encourage the adoption of patient DESIs and so it remains of interest to assess the outcomes using an implementation model; see Table 1. We do acknowledge that lack of detail and data made it difficult to assess the “degree of implementation” achieved; see Additional file 3, Table S2.

Judged against the implementation model, 10 of the 17 studies were categorized as achieving “insight” (see Additional file 3, Table S2), four achieved a level of “change” [26,28,34,36], and none of the studies indicated that organizations had been able to achieve “maintenance” levels, where DESIs were in sustained use. This may be due to the barriers identified in the studies, which contributed to recruitment patterns that showed low interest in participation and in less-than-anticipated distribution of these interventions to patients.

## Discussion

### Principal findings

Despite the increasing interest in moving patient decision support interventions from the world of randomized trials to that of routine settings, this review points to major implementation challenges. There are data indicating consistent positive patient outcomes, such as gain in knowledge, reported in efficacy trials [3]. However, despite these results, there is scarcely any evidence of sustainable adoption at organizational levels. The studies reported here paint a picture of professional indifference and organizational inertia, and where other priorities take precedence. Many of the barriers are similar to those encountered in other attempts to improve practice performance, where other competing priorities take precedence and where uncertainty about the added value of the proposed intervention favors the status quo [41]. Note that the organizations in these studies were willing volunteers and so implementation might be even more difficult in other settings. Although many countries are considering SDM in their policy developments, most of the implementation work to date has been located in North America. Ten studies were based in the US and three in Canada, illustrating the limited spread to areas beyond North America [9]. The majority of the work was conducted with limited resources in comparison to research funded by mainstream sources, such as the National Institutes for Health, and so in appraising these studies we need to recognize the constraints imposed by these limitations.

The studies do, however, reveal issues that are specific to the challenge of implementing patient DESIs. Reliance on clinicians to refer patients to these tools leads to limited utilization, and so using system-based approaches, where feasible, may help reach more patients. Unfortunately, system approaches rely on identifying eligible patients ahead of visits and this task is only possible for a limited number of conditions. Even when this is feasible, logistical and infrastructure challenges still impede integration into practice. When patients present with undifferentiated problems, identifying their decision support needs ahead of a visit may be impossible. This issue limits the scope for studies that adopt a referral model; most are based on clinical issues where prior identification is possible, e.g., invitations for screening and prevention. Yet, even in secondary care where it is often possible to predict the clinical decisions that will be needed, the process of ensuring patients use DESIs ahead of encounters is a challenge because the windows of opportunity are often short. Ultimately, the studies indicate that this degree of capital and logistical infrastructure is challenging to initiate and maintain and will require sustained investment [24,28,34,36,37,40]. These issues also make the limits of system-based approaches apparent and highlight the fact that referral by clinicians at the point of care will continue to be necessary for many clinical issues for which decision support is available.

All the included studies use a “referral model” of DESI dissemination whereby practitioners or their support staff identified patients eligible for decision support. The referral model proposes that these tools are “adjuncts” that support SDM, when used ahead of visits, or shortly afterwards [42]. However, the concept that these tools are positively viewed as “adjuncts” by clinicians does not seem to be supported in practice. Many of the studies report that professionals distrust the content of the tools, question their evidence-base, believe that they do not reflect “local” data, think that patients will decline to take part in decisions and, critically, that offering options is not what they would advocate from a “best practice” perspective. These findings suggest that the reluctance to prioritize the use of DESIs might lie deeper than a general resistance to change. The referral model might be based on assumptions about their contribution that is not shared by front-line clinicians [43], a suggestion we discuss further below. An alternative model where SDM is initiated by the practitioner in the space of clinical encounters, using briefer DESIs to catalyze dialogue about options, which in turn lead to the use of more extensive tools [44], does not seem to have been extensively investigated, although a few trials exist [45,46].

Although many barriers to implementation were described (Additional file 3, Table S2), these were seldom examined in depth, with the exception of three studies that employed qualitative interviews [23,34,25]. Additional insights might have been gained if more studies had explored the views of professionals regarding the use of DESIs and specifically about their impact on practice workflows.

### Strengths and weaknesses of the study method

The search strategy was developed in consultation with an information scientist and piloted before application to multiple electronic databases. We may have reported work as three separate studies [27,30,31] which should be viewed as a single study, but we did so because different methods were reported. We deliberately excluded work from conference proceedings and non-peer reviewed material and did not contact authors of included studies. The review does not attempt to pool the data from the studies nor assess their quality: the methods and results were too heterogeneous. The study team was experienced in the field and was familiar with the evidence-base. Dual independent review was accomplished at key stages of the review process. There was low inter-rater agreement on the first round of assessing implementation levels achievement and this required attention in a second round. The results were seldom organized in a way that assisted this assessment: more work is required to set clear criteria for assessing implementation attainment levels.

### Relation to other literature

The challenge of implementing patient DESIs is already well documented [1,2] and we also know that practitioners do not achieve SDM [47]. However, we must be careful not to equate the successful introduction of DESIs into clinical pathways as automatically leading to SDM. For instance, Frosch found that the use of a prostate specific antigen DESI ahead of a clinical encounter led to less SDM if a patient was not in favor of screening [48]. While we can be confident that these interventions have positive results at the patient level [3], we do not as yet fully understand their impact on clinician-patient dialogue. Other models where practitioners might use brief tools and take more responsibility for initiating the process of SDM face-to-face with patients deserve further investigation.

More use could have been made of developments in the evaluation of complex interventions [49], implementation, and evaluation studies [50]. Realist evaluations provide a way to study why interventions that have good effect in some settings fail when attempts are made to introduce them in other clinical settings: context matters [51]. Many opportunities exist to bring these worlds of inquiry to bear on how best to implement patient DESIs. Damschroder et al. provided a consolidated framework for advancing implementation science [50]: a synthesis of 19 models that describes five domains, namely, intervention characteristics, outer setting, inner setting, characteristics of the individuals involved, and the process of implementation. Future studies should consider the reported utility of these conceptual frameworks to guide implementation.

## Conclusions

The goal for this review was to make recommendations about how best to implement patient DESIs into practice. Having reviewed the existing studies, it seems too early for such recommendations. Perhaps the effort to implement was done too soon, ahead of any work done to achieve levels 1 and 2 of Grol’s model [21,22] – “orientation” and “insight” – in the recruited organizations. Without these first steps, it is unlikely that level 3, “acceptance”, would have occurred, and so the motivation to use patient DESIs might have been absent. Although it would not be difficult for us to suggest general principles of successful adoption [52], we feel that it might be more helpful to emphasize that the specific underlying issues that militate against the use of patient DESIs and, more generally, limit the adoption of SDM, are under-investigated and under-specified.

However, we do have two substantive research recommendations. It would be helpful to have a framework for reporting these studies, based on the SQUIRE guidelines [53], adapted to cover the reporting of the patient identification processes, the numbers of patients eligible for specific DESIs (initial denominator), the inevitable attrition in numbers along the delivery pathway, the delivery mechanism, the evaluation of use by the patient and the impact on decision outcomes (process and quality). In addition, approaches not previously used in this field should be considered as a means to investigate and measure the challenges of implementing new delivery-systems [54]. For instance, methods such as cognitive task analysis, ethnography and action research, tools to assess the “adaptive reserve’” of teams [55] or their “readiness for change” [56], are approaches that would pay more attention to the role of the participants in shaping and using the technologies [57], and how they fit into the demands of other technologies, such as the electronic medical record and demands for performance metrics. Amidst all of this will be the need to monitor which professional and team-related behaviors will be rewarded as health systems increasingly seek to ensure patients experience better quality of care [58]. As a final comment, we need to acknowledge that all the existing studies operated in a policy context where no rewards or incentives existed to promote the use of patient decision support and were being done in parallel in a period where considerable resources were being invested in the adoption of electronic health care records. Paraphrasing Robert Frost, there are many miles to go before we sleep.

## Competing interests

The Informed Medical Decisions Foundation (IMDF) is a not-for-profit (501(c)(3)) private foundation that develops content for patient decision support tools and has a royalty arrangement with a for-profit company, Health Dialog. The tools are used as part of the services Health Dialog provides to consumers through health care organizations and employers. Glyn Elwyn, Carmen Lewis, and Dominick Frosch have received research funding from IMDF. Glyn Elwyn and Dominick Frosch have received travel support and honoraria from IMDF, and Richard Wexler receives salary as a Director from IMDF. Isabelle Scholl, Caroline Tietbohl, Mala Mann, Adrian Edwards, Catharine Clay, France Légaré, and Trudy van der Weijden declare that they have no competing interests.

## Authors’ contributions

GE and DF designed the review with support from MM, and IS; and AE. GE, DF, IS, CT, and MM were responsible for the search, selection and extraction processes. All other authors contributed to the review protocol and the coordination of the work. All authors read, contributed equally to, and approved the final manuscript.

## Supplementary Material

Additional file 1 **Appendix 1:** Search strategy Click here for file

Additional file 2 **Table S1:** Summaries of patient decision support implementation studies: Aims, implementation strategies, outcomes Click here for file

Additional file 3 **Table S2:** Reported barriers, facilitators, and levels of implementation Click here for file

## Declarations

No direct funding was received from any source to support this review.

Publication of this supplement was partially funded by an unrestricted grant from the Informed Medical Decisions Foundation. The remaining funding came from Trudy van der Weijden; the Department of Medical Psychology, Hamburg (Isabelle Scholl); and the Dartmouth Center for Health Care Delivery Science (Glyn Elwyn). Administrative and editorial support for this supplement was also provided by The University of Texas MD Anderson Cancer Center.

This article has been published as part of *BMC Medical Informatics and Decision Making* Volume 13 Supplement 2, 2013: The International Patient Decision Aid Standards (IPDAS) Collaboration’s Quality Dimensions: Theoretical Rationales, Current Evidence, and Emerging Issues. The full contents of the supplement are available online at http://www.biomedcentral.com/bmcmedinformdecismak/supplements/13/S2.
