# Supplementary material for: “Many miles to go …”: a systematic review of the implementation of patient decision support interventions into routine clinical practice
Source: BMC Med Inform Decis Mak. 2013 Nov 29;13(Suppl 2):S14. doi: 10.1186/1472-6947-13-S2-S14 (PMC4044318; doi:10.1186/1472-6947-13-S2-S14)
Supplement: Additional file 1 — Appendix 1: Search strategy [file 1472-6947-13-S2-S14-S1.pdf]

Appendix 1: Search strategy

1. exp Decision Making/ (95638)
2. exp Patient Participation/ (14669)
3. (share* adj decision adj mak*).ti,ab. (1164)
4. (decis* adj mak*).mp. (97836)
5. (decis* adj choic*).tw. (42)
6. shared decision making.mp. (1142)
7. (patient adj3 decision making).tw. (1211)
8. or/1-7 (146942)
9. exp Decision Support Techniques/ (46924)
10. exp Decision Support Systems, Clinical/ (3461)
11. decision making techniques.tw. (36)
12. decision support method*.tw. (34)
13. (decision adj3 aids).tw. (592)
14. decision support intervention*.tw. (48)
15. decision making strat*.tw. (224)
16. decision making approach*.tw. (110)
17. or/9-16 (50838)
18. exp Organizational Culture/ (10369)
19. (embed* or set in or rooted).tw. (65537)
20. (integrat* adj3 organi?ational).tw. (159)
21. "put into practice".tw. (544)
22. (routine adj3 (care or practice)).mp. (11645)
23. organi?ational system.mp. (89)
24. (apply or application or utili?e or utili?ation or usage).tw. (544512)
25. (disseminat* or implement*).mp. (228214)
26. or/18-25 (832290)
27. (implement* adj3 patient decision*).mp. (11)
28. (implement* adj3 decision aid*).mp. (27)
29. (appl* adj3 decision aid*).mp. (12)
30. (utili?* adj3 decision aid*).mp. (5)

31. or/27-30 (48)
32. 8 and 17 and 26 (1259)
33. 31 or 32 (1279)
