# Supplementary material for: “Many miles to go …”: a systematic review of the implementation of patient decision support interventions into routine clinical practice
Source: BMC Med Inform Decis Mak. 2013 Nov 29;13(Suppl 2):S14. doi: 10.1186/1472-6947-13-S2-S14 (PMC4044318; doi:10.1186/1472-6947-13-S2-S14)
Supplement: Additional file 2 — Table S1: Summaries of patient decision support implementation studies: Aims, implementation strategies, outcomes [file 1472-6947-13-S2-S14-S2.pdf]

Table S1. Summaries of patient decision support implementation studies: aims, implementation strategies, outcomes

**Author, year, study citation, country.**<br><br>**Setting: study type.**<br><br>**Funding.**	**Study aims. Conceptual framework.**	**Intervention and implementation strategy.**<br><br>**Study duration.**	**Implementation and provider outcomes.**
Holmes-Rovner, 2000, [1], US.<br><br>Secondary care hospital: observational study.<br><br>Funding: Blue Cross and Blue Shield health insurers.	To determine feasibility of using DESIs in a fee-for-service hospital system (physicians' offices and in-patient facilities).<br><br>Framework: nil reported.	Invited hospitals in Michigan to participate in evaluation of DESI implementation. 5 hospitals selected based on interest, physician leadership, patient volumes, and evidence of supportive staff and systems.<br><br>8 months: pilot October–December 1996; initial implementation period January–May 1997.	Estimated potential eligible patients across all sites per year: 1319 breast cancer, 1222 ischemic heart disease. Use of DESIs was lower than anticipated: 25 patients referred over 7 months in 3 hospitals; 4 of 27 eligible breast cancer patients referred at one site. Number of eligible and referred patients not reported for ischemic heart disease.
Stapleton, 2002, [2], UK.<br><br>Secondary care maternity units: quasi-experimental and observational study.<br><br>Funding: Department of Health.	To evaluate use and impact of evidence-based leaflets on informed choice in maternity services.<br><br>Framework: nil reported.	13 maternity units allocated to 10 clusters: 5 intervention clusters that received DESIs (leaflets) were compared to 5 controls.<br><br>Study duration not reported.	70% of women recruited reported receiving at least one leaflet. Health professionals rarely observed discussing leaflets with patients.
Stacey, 2005, [3], Canada.<br><br>Call-centers:	To evaluate barriers and facilitators influencing provision of decision support by call-center	Using simulated callers, evaluated performance of call-center nursing staff introduced to patient decision support. A survey, semi-structured	99 nurses were approached and 30 consented to undertake evaluation by simulated calls. During these calls, 28 nurses used decision support to

observational study.<br><br>Funding: sources related to Canada research Chair.	nurses.<br><br>Framework: Ottawa model of research use and Ottawa Decision Support.	interviews, and focus groups were used to collect data.<br><br>2 months: December 2003–January 2004.	varying extents.
Silvia, 2006, [4], US.<br><br>Secondary care oncology: observational study.<br><br>Funding: IMDF.	To characterize patterns of use and perceived barriers to implementation in clinical sites that had shown expressed interest in providing patient DESIs.<br><br>Framework: nil reported.	Convenience samples of 15 US sites identified and informed about availability of DESIs. Each site was sent 10 copies (DVDs). Follow-up interviews scheduled at 6-12 months.<br><br>DESIs allocated January–August 2003. Total duration not reported.	All 15 sites requested additional DVDs. 6 of 15 sites implemented DESIs and 9 were willing to be interviewed. Patient exposure to the DESI varied. 5 sites reported reaching between 40% t0 80% of eligible patients.
Stacey, Pomey et al., 2006, [5], Canada.<br><br>Call-center: case study.<br><br>Funding: Canadian Institute for Health Research.	To describe and evaluate experience interacting with a nurse-staffed call-center using a decision support protocol.<br><br>Framework: Ottawa model of research use.	Provided online tutorial, skills workshop, provision of a decision support protocol, and feedback on quality of decision support provided to pre-arranged simulated calls.<br><br>7 months: December 2003–June 2004.	31 nurses received the intervention, 25 responded to a survey. Majority of nurses reported positive future intentions to use decision support protocol. A month after the workshop, 11 of the 25 reported actual use.
Stacey, O'Connor et al., 2006, [6], Canada.<br><br>Call-center: RCT.<br><br>Funding: Ontario Ministry of Health.	To evaluate implementation of decision support and decision coaching in a nurse-led call-center.<br><br>Framework: nil reported.	Provided education regarding coaching and skills workshop. Baseline and post-intervention data collected using calls by simulated patients.<br><br>Second simulated call one month after intervention. Total duration not reported.	Compared with controls (n=20), nurses in intervention group (n-21) had greater knowledge and improved decision coaching skills. Provision of decision support did not increase call duration.

References

[1] Holmes-Rovner M V, Valade D, Orlowski C, Draus C, Nabozny-Valerio B, Keiser S: **Implementing shared decision-making in routine practice: barriers and opportunities**. *Health Expect* 2000, **3**:182–191.

[2] Stapleton H, Kirkham M, Thomas G: **Qualitative study of evidence based leaflets in maternity care**. *BMJ* 2002, **324**:639.

[3] Stacey D, Graham I, O'Connor A, Pomey M: **Barriers and facilitators influencing call center nurses' decision support for callers facing values-sensitive decisions: a mixed methods study**. *Worldv Evid-Based Nu* 2005, **2**:184–195.

[4] Silvia KA, Sepucha KR: **Decision aids in routine practice: lessons from the breast cancer initiative**. *Health Expect* 2006, **9**:255–264.

[5] Stacey D, Pomey M, O'Connor A, Graham I: **Adoption and sustainability of decision support for patients facing health decisions: an implementation case study in nursing**. *Implement Sci* 2006, **1**:17.

[6] Stacey D, O'Connor AM, Graham ID, Pomey MP: **Randomized controlled trial of the effectiveness of an intervention to implement evidence-based patient decision support in a nursing call centre**. *Implement Sci* 2006, **12**:410–415.
