# Supplementary material for: “Many miles to go …”: a systematic review of the implementation of patient decision support interventions into routine clinical practice
Source: BMC Med Inform Decis Mak. 2013 Nov 29;13(Suppl 2):S14. doi: 10.1186/1472-6947-13-S2-S14 (PMC4044318; doi:10.1186/1472-6947-13-S2-S14)
Supplement: Additional file 3 — Table S2: Reported barriers, facilitators, and levels of implementation [file 1472-6947-13-S2-S14-S3.pdf]

Table S2. Reported barriers, facilitators, and levels of implementation

**Author, year, study citation, country.**<br><br>**Setting: study type.**<br><br>**Funding.**	**Identified barriers**	**Identified facilitators**	**Level of implementation achieved**<br>1. Orientation<br>2. Insight<br>3. Acceptance<br>4. Change<br>5. Maintenance
Holmes-Rovner, 2000, [1], US.<br><br>Secondary care hospital: observational study.<br><br>Funding: Blue Cross and Blue Shield health insurers.	DESI provision not integrated into role or task expectations, i.e. clinicians 'forgot' to give tools to patients. Logistical challenges also reported, e.g. collection of tools from a separate center and rapid scheduling of patients for surgery did not provide time for patients to consider decisions fully. Professional skepticism about value of decision support was reported and accounts that tools were viewed as good sources of information but to involve patients in decisions.	Not reported.	2. Insight.

Stapleton, 2002, [2], UK.<br><br>Secondary care maternity units: quasi-experimental and observational study.<br><br>Funding: Department of Health.	Competing demands in clinical environments, time pressures, clinical unavailability of some treatments (leaflets described options that were not available locally) and staff disagreement with leaflet content, hierarchical professional power structures, where obstetricians defined "right" choices, failure to distinguish leaflets from other information related to pregnancy, packaging of leaflets in advertising or maternity folders, failure to understand shared decision making and lack of continuity of care during pregnancy.	Not reported.	2. Insight.
Stacey, 2005, [3], Canada.<br><br>Call-centers: observational study.<br><br>Funding: sources related to Canada research Chair.	Difficulty in using DESIs via telephone, lack of ability and confidence to address callers' decisional needs, increased call length, and a lack of knowledge regarding available health services within the caller's community. Organizational factors: e.g. pressure to minimize call length, novelty of providing decision support at a call center, and lack of performance standards.	Prior nursing experience of patient decision support. Existence of tailored call-center infrastructure.	2. Insight.

Silvia, 2006, [4], US.<br><br>Secondary care oncology: observational study.<br><br>Funding: IMDF.	Lack of clinical motivation to use DESIs and reported shortage of time and resources. Logistical challenge of providing patients with time and space to view DVDs. Decision support was viewed as being in competition with other existing patient information. Concerns about ‘overwhelming’ patients.	Existence of a clinical champion, especially when in a leadership position. Systematic approach for integrating provision and use of patient DESIs support into clinical pathways.	2. Insight.
Stacey, Pomey et al., 2006, [5], Canada.<br><br>Call-center: case study.<br><br>Funding: Canadian Institute for Health Research.	Difficulty in using decisions support materials over telephone. Concern that call efficiency might be compromised. Perceived inadequate skills and low confidence in providing decision support.	The provision of training and introduction of a patient decision support protocol.	2. Insight.
Stacey, O’Connor et al., 2006, [6], Canada.<br><br>Call-center: RCT.<br><br>Funding: Ontario Ministry of Health.	Not reported.	Not reported.	2. Insight.
Garden, 2008, [7],UK.<br><br>Secondary care urology clinics: observational study.<br><br>Funding: Department of Health, UK.	No barriers reported.	Not reported.	2. Insight.
Silvia, 2008, [8], US.	Lack of clinician support for using DESI, due to lack of time and unfamiliarity with	Accepting added value of using DESIs facilitated implementation: more patients	2. Insight.

Secondary care oncology: observational study.<br><br>Funding: IMDF	content. The resistance of other professionals, e.g. nursing staff, also reported, specifically due to a concern that patients resist engaging in decisions at a time of cancer diagnosis.	received and used DESIs when nurses were involved in recommending their use.	
Stacey, 2008, [9], Australia.<br><br>Cancer helpline service: pre- and post-assessment.<br><br>Funding: not reported.	Limited awareness of patient decision support, potential organizational ambivalence for the task, and low confidence in new specific skills.	Positive attitudes to patient involvement in decision-making, having sufficient time for more complex calls, the provision of training and orientation.	2. Insight.
Belkora, 2009, [10], US.<br><br>Secondary care breast care: case study.<br><br>Funding: IMDF.	Costs of producing and distributing decision support, lack of infrastructure for patients to view DESIs, lack of patient access to telephones, and delivery of decision support was not integrated into role or task expectations.	Re-engineer pathway so that viewing DVDs and decision coaching is provided to eligible patients prior to clinical encounters.	3. Acceptance.
Brackett, 2010, [11], US.<br><br>Primary care, rural academic medical centers: quasi-experimental study.<br><br>Funding: IMDF.	Distribution failed due to clinicians being 'distracted by other duties'. Identification of eligible patients and referral of patients to DESIs are not part of existing routines.	Systematize the distribution of patient DESIs. This is made easier when categories of patients can be identified, e.g. due for screening or preventative visits.	4. Change.

Belkora, 2011, [12], US.<br><br>Secondary care: quality improvement study.<br><br>Funding: IMDF.	Delivery of decision support was not integrated into role or task expectations.	Telephone delivery of decision coaching and flexible scheduling to maximize use of marginal staff time. Removing the task of identifying patients eligible for decision support from clinical roles. Systematize the distribution of patient DESI– for example, use mail rather than use referral dependent loan services.	4. Change.
Feibelmann, 2011, [13], US.<br><br>Mixed care settings for breast cancer: quasi-experimental study.<br><br>Funding: IMDF.	Difficulty identifying eligible patients. Lack of time and resources contribute to the logistical challenge of distributing DESIs. The diversity and volume of other existing educational materials. Resistant professional attitudes reported: e.g. lack of 'trust' in DESI content and design, and a view that patients lack sufficient literacy and 'do not want' to be involved in decisions.	Not reported.	4. Change (28 of 111 sites).
Holmes-Rovner, 2011, [14], US.<br><br>Primary care internal/family medicine clinics: observational study.<br><br>Funding: IMDF.	No barriers reported.	Clinician skill development using simulations and reimbursement for undertaking shared decision making.	3. Acceptance.

Frosch, 2011, [15], US.<br><br>Primary care practices: observational study.<br><br>Funding: IMDF	Lack of adequate infrastructure, inefficient identification of eligible patients, work environment tensions among physicians and support staff, patient frustration due to long wait times, staff juggling competing demands, disinterested physicians.	Efficient infrastructure; efficient identification of eligible patients; good rapport between physicians, staff, and patients; interested motivated professionals who provide 'warm hand-offs' as they refer patients to DESIs.	3. Acceptance.
Miller, 2011, [16], US.<br><br>Academic internal medicine practice: observational study.<br><br>Funding: IMDF	Difficulty identifying eligible patients, infrastructure required for DVD viewing and time needed view DESIs in clinic.	Not reported.	4. Change.
Uy, 2011, [17], US.<br><br>Primary care practices: observation, qualitative.<br><br>Funding: IMDF	Scare workforce capacity, competing clinical demands, language barriers (DESIs only available in English), clinician perception of patient resistance to DESIs, low levels of staff interest.	Lead physician engagement and buy-in. Other facilitators: DESI storage and accessibility, clear lists of available DESI, content summaries for use by staff, posters advertising the availability of DESIs to patients.	2. Insight.

References

[1] Holmes-Rovner M V, Valade D, Orlowski C, Draus C, Nabozny-Valerio B, Keiser S: **Implementing shared decision-making in routine practice: barriers and opportunities**. *Health Expect* 2000, **3**:182–191.

[2] Stapleton H, Kirkham M, Thomas G: **Qualitative study of evidence based leaflets in maternity care**. *BMJ* 2002, **324**:639.

[3] Stacey D, Graham I, O'Connor A, Pomey M: **Barriers and facilitators influencing call center nurses' decision support for callers facing values-sensitive decisions: a mixed methods study**. *Worldv Evid-Based Nurs* 2005, **2**:184–195.

[4] Silvia KA, Sepucha KR: **Decision aids in routine practice: lessons from the breast cancer initiative**. *Health Expect* 2006, **9**:255–264.

[5] Stacey D, Pomey M, O'Connor A, Graham I: **Adoption and sustainability of decision support for patients facing health decisions: an implementation case study in nursing**. *Implement Sci* 2006, **1**:17.

[6] Stacey D, O'Connor AM, Graham ID, Pomey MP: **Randomized controlled trial of the effectiveness of an intervention to implement evidence-based patient decision support in a nursing call centre**. *Implement Sci* 2006, **12**:410–415.

[7] Garden J: **Implementing an informed decision making programme for urology patients**. *J Commun Healthc* 2008, **1**:297–310.

[8] Silvia K, Ozanne E, Sepucha K: **Implementing breast cancer decision aids in community sites: barriers and resources**. *Health Expectat* 2008, **11**:46–53.

[9] Stacey D, Chambers SK, Jacobsen MJ, Dunn J: **Professionals providing decision support for callers : an implementation study**. *Oncol Nurs Forum* 2008, **35**:961–969.

[10] Belkora JK, Loth MK, Volz S, Rugo HS: **Implementing decision and communication aids to facilitate patient-centered care in breast cancer: a case study.** *Patient Educ Couns* 2009, **77**:360–368.

[11] Brackett C, Kearing S, Cochran N, Tosteson A, Blair Brooks W: **Strategies for distributing cancer screening decision aids in primary care**. *Patient Educ Couns* 2010, **78**:155–16.

[12] Belkora JK, Teng A, Volz S, Loth MK, Esserman LJ: **Expanding the reach of decision and communication aids in a breast care center: a quality improvement study.** *Patient Educ Couns* 2011, **83**:234–239.

[13] Feibelmann S, Yang TS, Uzogara EE, Sepucha K: **What does it take to have sustained use of decision aids? A programme evaluation for the Breast Cancer Initiative.** *Health Expect* 2011, **14 Suppl 1**:85–95.

[14] Holmes-Rovner M, Kelly-Blake K, Dwamena F, Dontje K, Henry RC, Olomu A, Rovner DR, Rothert ML: **Shared Decision Making Guidance Reminders in Practice (SDM-GRIP)**. *Patient Educ Couns* 2011, **85**:219–24.

[15] Frosch DL, Singer KJ, Timmermans S: **Conducting implementation research in community-based primary care: a qualitative study on integrating patient decision support interventions for cancer screening into routine practice**. *Health Expect* 2011, **14**:73–84.

[16] Miller KM, Brenner A, Griffith JM, Pignone MP, Lewis CL: **Promoting decision aid use in primary care using a staff member for delivery.** *Patient Educ Couns* 2011, **86**:189–94.

[17] Uy V, May SG, Tietbohl C, Frosch DL: **Barriers and facilitators to routine distribution of patient decision support interventions: a preliminary study in community-based primary care settings.** *Health Expect* 2012, **Epub ahead**:1–12.
